# 

**Published:** 1856-06

**Authors:** 


### TO CONTRIBUTORS.

We return our thanks for a number of communications received recently, all of which will appear in due time. We must still, however, urge our friends to frequent contributions, as upon our original department mainly depends the permanent prosperity of the Journal.

Our hopes in reference to the position which this Journal would occupy, have been fully realized, and we have the less hesitation in making the statement, as we know it to be mainly attributable to our contributors.

### TO CORRESPONDENTS.

☞ All Communications pertaining to the Editorial Department of the Journal, should be addressed to the Editors.

☞ All Communications, having reference to the Subscription, the Advertising or the Financial Department, should be addressed to the Treasurer, J. G. WESTMORELAND, M. D., Dean of the Faculty of the Atlanta Medical College.

---

☞ Messrs. A. TILDEN and W. H. PHILLPOT are authorized Agents for this Journal.

